# Management of pineal region tumors in a pediatric case series

**DOI:** 10.1007/s10143-020-01323-1

**Published:** 2020-06-06

**Authors:** Matthias Schulz, Melissa Afshar-Bakshloo, Arend Koch, David Capper, Pablo Hernáiz Driever, Anna Tietze, Arne Grün, Ulrich-Wilhelm Thomale

**Affiliations:** 1 Pediatric Neurosurgery, Charité-Universitätsmedizin Berlin, Corporate Member of Freie Universität Berlin, Humboldt-Universität zu Berlin, and Berlin Institute of Health, Augustenburger Platz 1, 13353 Berlin, Germany; 2 Department of Neuropathology, Charité-Universitätsmedizin Berlin, Corporate Member of Freie Universität Berlin, Humboldt-Universität zu Berlin, and Berlin Institute of Health, Berlin, Germany; 3 Department of Pediatric Oncology and Hematology, Charité-Universitätsmedizin Berlin, Corporate Member of Freie Universität Berlin, Humboldt-Universität zu Berlin, and Berlin Institute of Health, Berlin, Germany; 4 Institute of Neuroradiology, Charité-Universitätsmedizin Berlin, Corporate Member of Freie Universität Berlin, Humboldt-Universität zu Berlin, and Berlin Institute of Health, Berlin, Germany; 5 Department of Radiation Oncology, Charité-Universitätsmedizin Berlin, Corporate Member of Freie Universität Berlin, Humboldt-Universität zu Berlin, and Berlin Institute of Health, Berlin, Germany

**Keywords:** Pineal region tumor, Neuroendoscopy, Biopsy, ETV, Shunt

## Abstract

Pineal region tumors commonly present with non-communicating hydrocephalus. These heterogeneous histological entities require different therapeutic regimens. We evaluated our surgical experience concerning procurance of a histological diagnosis, management of hydrocephalus, and choice of antitumoral treatment. We analyzed the efficacy of neuroendoscopic biopsy and endoscopic third ventriculocisternostomy (ETV) in patients with pineal region tumors between 2006 and 2019 in a single-center retrospective cross-sectional study with regard to diagnostic yield, hydrocephalus treatment, as well as impact on further antitumoral management. Out of 28 identified patients, 23 patients presented with untreated hydrocephalus and 25 without histological diagnosis. One patient underwent open biopsy, and 24 received a neuroendoscopic biopsy with concomitant hydrocephalus treatment if necessary. Eighteen primary ETVs, 2 secondary ETVs, and 2 ventriculoperitoneal shunts (VPSs) were performed. Endoscopic biopsy had a diagnostic yield of 95.8% (23/24) and complication rates of 12.5% (transient) and 4.2% (permanent), respectively. ETV for hydrocephalus management was successful in 89.5% (17/19) with a median follow-up of more than 3 years. Following histological diagnosis, 8 patients (28.6%) underwent primary resection of their tumor. Another 9 patients underwent later-stage resection after either adjuvant treatment (*n* = 5) or for progressive disease during observation (*n* = 4). Eventually, 20 patients received adjuvant treatment and 7 were observed after primary management. One patient was lost to follow-up. Heterogeneity of pineal region tumor requires histological confirmation. Primary biopsy of pineal lesions should precede surgical resection since less than a third of patients needed primary surgical resection according to the German pediatric brain tumor protocols. Interdisciplinary decision making upfront any treatment is warranted in order to adequately guide treatment.

## Introduction

Tumors of the pineal region constitute only 2.8–11% of all brain tumors in children and adolescents and yet represent a challenge in terms of diagnosis and treatment [24]. This is not only due to the high diversity of tumor entities including pineal parenchymal tumors, germ cell tumors (GCTs), and tumors arising from neuroectodermal tissue as major subgroups but also due to the deep-seated anatomical location and proximity to eloquent structures [22, 32, 35]. Moreover, the anatomical relation of the pineal gland to the aqueduct and the risk of obstruction by a space-occupying mass dispose patients to non-communicating hydrocephalus as a presenting clinical sign, which needs to be considered for the surgical strategy. Depending on the histological diagnosis, not all patients may require surgical resection as first-line treatment. Thus, as a first therapeutic step, treatment of the non-communicating hydrocephalus may be performed along with an establishment of the histological diagnosis to guide further management. Surgical options for biopsy are a stereotactic, endoscopic, or open microsurgical approach, while only the latter two options enable concomitant hydrocephalus treatment. Moreover, the combination of a typical tumorous lesion of the pineal region and the laboratory findings of elevated alpha fetoprotein (AFP) and beta human choriogonadotropin (β-HCG) in either serum or cerebrospinal fluid establishes the diagnosis of a secreting germ cell tumor. In this situation, according to the respective guidelines, no further histological confirmation is necessary [5]. Nevertheless, secreting mixed germ cell tumors do represent complex neoplasms which may warrant surgical therapy during the course of treatment.

Of the possible options, the neuroendoscopic approach may be advantageous to simultaneously obtain a histological diagnosis and to treat non-communicating hydrocephalus if present and has evolved to be our preferred approach to a newly diagnosed pineal region tumor. The aim of the current retrospective study was to evaluate (a) the efficacy of the selected neuroendoscopic biopsy with regard to its diagnostic yield and accuracy and (b) the course of the subsequent therapeutic management (surgical resection vs. observation vs. chemotherapy/radiation therapy) once the histology of the pineal region tumor was confirmed.

## Patients and methods

We performed a retrospective cross-sectional analysis of our database of the pediatric neurosurgical department to identify all patients with tumors located in the pineal region and those who underwent surgical treatment between January 2006 and January 2019. The medical records, surgical notes, and all available radiological imaging were reviewed to identify presenting symptoms, type of surgical procedure, possible complications, histological findings, and further surgeries during follow-up. Furthermore, all available information about adjuvant chemotherapy and/or radiation therapy and patients’ clinical condition at the time of final analysis was collected. Retrospective data collection was approved by the local ethics committee.

### Technique of biopsy

**Fig. 1:**
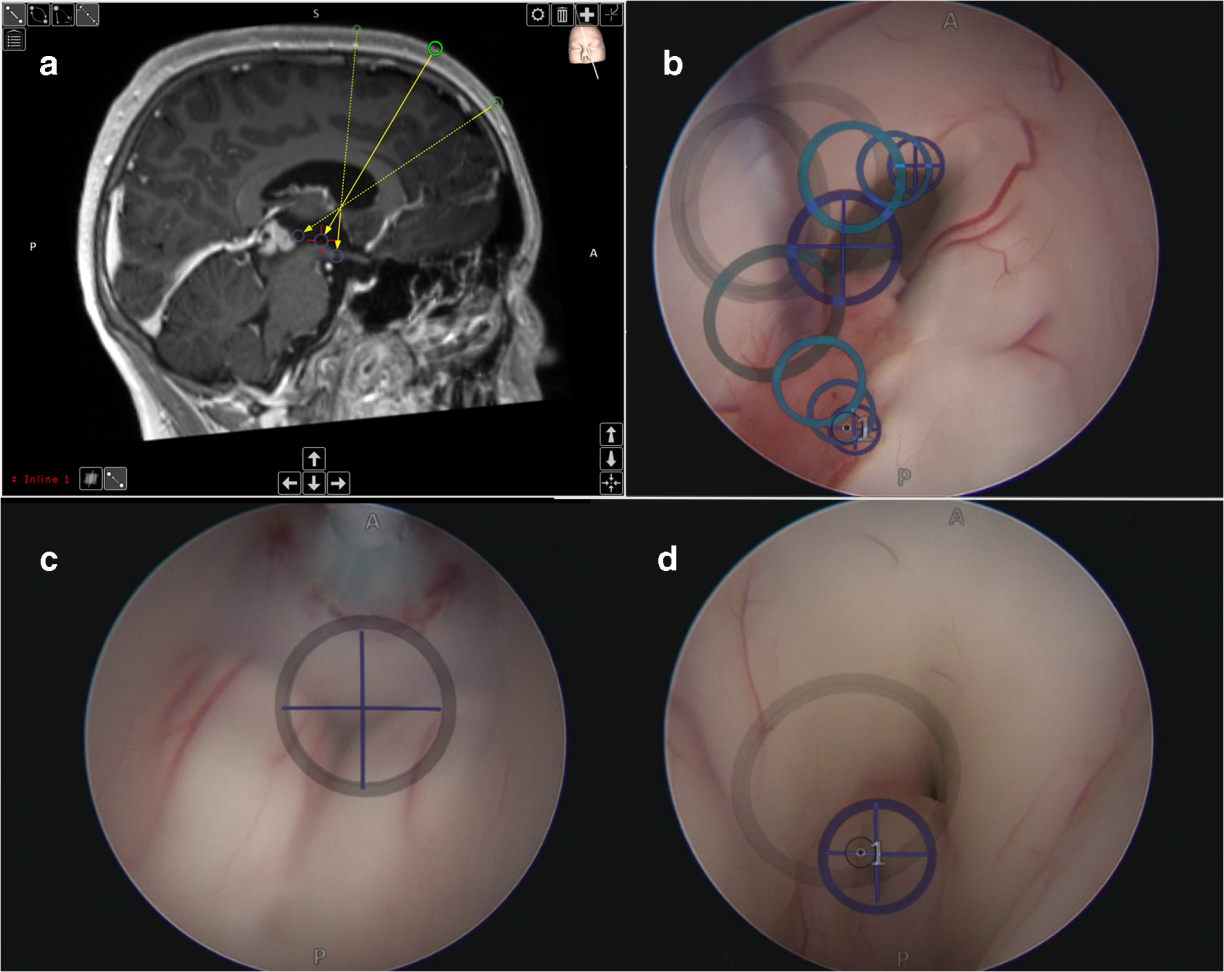
Neuroendoscopic biopsy and third ventriculostomy. **a** Planning of two *ideal* trajectories, both through the foramen of Monro to the floor of the third ventricle and the pineal region tumor. The bisecting trajectory is the resulting trajectory into the ventricular system. **b** Intraoperative view of the right foramen of Monro with the superimposed trajectories to the floor and the pineal region. **c** Performing of an endoscopic third ventriculostomy. **d** Intraoperative view of the pineal region tumor (histology: germinoma) with superimposed preplanned target area (augmented reality—SCOPIS Neuro Navigation, Stryker)

Specifically, the techniques for initial biopsy as well as the type and success of hydrocephalus treatment were evaluated. If an endoscopic treatment for hydrocephalus was indicated, it was mainly aimed to acquire a biopsy in the same procedure as well. Since mostly endoscopic biopsy was performed simultaneously to an endoscopic third ventriculostomy (ETV) via a single burr hole, the surgical technique is briefly described. To define the burr hole localization to reach both targets without major manipulation at the level of the foramen of Monro, the mean entry point between both optimal entries for the floor of the third ventricle and the tumor within the third ventricles was calculated. Therefore, the targets were connected through the foramen of Monro and the resulting trajectory was elongated towards the skull convexity. Thus, an anterior entry point was defined to reach the tumor and a posterior, pericoronal entry point was defined to be optimal to reach the floor of the third ventricle. Calculating the exact midpoint between both entry points on the convexity results in the optimal single-burr hole localization to reach both targets with a rigid endoscope [8, 16]. Navigated endoscopy enabled accurate planning as well as accurate execution of the procedure. Since the endoscope (Minop, Aesculap, Germany) includes the working channel anterior to the optic, it was introduced in the third ventricle in opposite orientation in order to bring the working channel as close as possible towards the target, respectively (Fig. 1).

### Treatment protocol

All patients were registered and treated along treatment protocols that included chemotherapy and/or irradiation and were approved by the German Society of Pediatric Oncology and Hematology (GPOH) and the German “Hirntumornetzwerk für Kinder und Jugendliche” (HIT). The respective treatment protocols are given as indicated. The indication for surgical resection was determined by a multidisciplinary team decision depending on the specific histological diagnosis according to its respective treatment protocol as either primary treatment (teratoma, low-grade glioma), before chemotherapy (high-grade glioma), or after neoadjuvant chemotherapy (malignant or mixed germ cell tumor, malignant pineal parenchymal tumor). No surgical resection is indicated in pure germinoma.

The extent of resection was graded as gross total resection (GTR, no obvious residual on postoperative magnetic resonance imaging (MRI)), subtotal resection (STR, maximal residual tumor less than 1.5 cm^2^), partial resection (PR, residual tumor larger than 1.5 cm^2^), and extended biopsy (no significant change in tumor size).

The stage of treatment outcome at the time of last follow-up was evaluated as complete remission (CR), stable disease (SD), progressive disease (PD), or death.

## Results

We identified a total of 28 pediatric patients (13 females) with a pineal region tumor that were surgically treated at the pediatric neurosurgical department at Charité-Universitätsmedizin Berlin. The median age at diagnosis was 12 years and 4 months (range 5 months to 17 years and 5 months) with a median follow-up of 4 years and 1 month (range 1 month to 12 years).

At the time of first referral to our department, the histological diagnosis was established in 3 patients (10.7%; one pilocytic astrocytoma (PA), one mixed malignant germ cell tumor, and one choriocarcinoma) via stereotactic, open, and endoscopic biopsy procedures at the referring hospital, respectively. For the remaining 25 patients (89.3%), no previous diagnostic intervention was performed. At the time of first presentation, 23 patients (82.1%) had clinical and radiological signs of non-communicating hydrocephalus. At referral, two patients were already treated with implantation of a ventriculoperitoneal shunt (VPS). Of those, one patient received the VP shunt previously during infancy due to posthemorrhagic hydrocephalus; the pineal region tumor was identified during routine MRI. The other patient was treated acutely with significant symptoms of non-communicating hydrocephalus caused by the pineal region mass. All other patients underwent a diagnostic MRI because of newly developed symptoms that finally led to the diagnosis of a pineal lesion. Clinical symptoms consisted of headaches in 19 of 25 (76.0%), nausea and vomiting in 18 of 25 (72.0%), gaze palsy in 12 of 25 (48.0%), impaired visual acuity 7 of 25 (28.0%), and motor symptoms or ataxia 5 of 15 (20.0%).

### Primary surgical intervention

All patients without preexisting histological diagnosis (*n* = 25) underwent primarily a biopsy of their lesion, which was performed along with concomitant treatment of the non-communicating hydrocephalus if present. For 12 patients, preoperative serum and CSF levels of pineal tumor markers (AFP and β-HCG) were collected. Of those, 10 were negative, but elevated in 2 patients. In 6 patients, only serum levels of AFP and β-HCG were available, which were within the normal range, while in 7 patients, no preoperative tumor markers were available. Out of 25 patients, two patients without hydrocephalus were AFP and β-HCG negative in serum and CSF but confirmed to have a germinoma by biopsy. All other patients presented with non-communicating hydrocephalus in whom a surgical intervention was indicated. Of those, two patients with elevated CSF levels of AFP and β-HCG were histologically diagnosed as malignant teratoma and mixed malignant germ cell tumor, respectively.

An initial ETV together with endoscopic biopsy was performed in 14 patients, an ETV with placement of a stent through the floor of the third ventricle into the prepontine cistern (stented ETV (sETV)) [34] and endoscopic biopsy in 4 patients, while the placement of an external ventricular drain (EVD) and endoscopic biopsy were done in 2 patients. The placement of a VPS and endoscopic biopsy were performed in 2 patients (one infant and one tumor occluding the third ventricle), an endoscopic biopsy alone was performed in 2 patients, and a parietal craniotomy with transventricular approach led to open biopsy in 1 patient. The latter patient was the first patient of this series.

### Diagnostic and accuracy rate of biopsy

Biopsies of the pineal lesion were successful to establish a histological diagnosis in 24 of 25 patients (96.0%). Excluding the open biopsy via craniotomy, the success rate of endoscopic biopsy to yield a histological result was 23 of 24 (95.8%). For one patient (4.2%) with non-diagnostic endoscopic biopsy but successfully treated hydrocephalus, a stereotactic biopsy was needed, which established a histological diagnosis.

For the newly biopsied pineal lesions, histological diagnoses according to the WHO classification of central nervous system tumors were as follows: 4 pineal parenchymal tumors (1 papillary tumor of the pineal region, 1 pineal anlage tumor, 2 pineoblastoma), 8 GCTs (4 germinoma, 3 mixed malignant germ cell tumor, 1 malignant teratoma), 12 glial tumors (2 ganglioglioma, 2 pilocytic astrocytoma, 2 low-grade astroglial tumor, 1 diffuse astrocytoma, 3 anaplastic astrocytoma, 1 H3K27-mutated midline glioma, 1 glioblastoma multiforme), and 1 ependymal cyst (Table 1).

**Table 1:** Tumor histology, biopsy, and patient outcome

Group	Entity	Biopsy	ETV	Outcome
Pineal Parenchymal Tumors [n=4, 14%]	papillary tumor of the pineal region	1	1	SD n=1
	pinealisanlagentumor	1	-	SD n=1
	pineoblastoma	2	2 [1 shunted]	SD n=1; dead n=1
Germ Cell Tumors (GCT) [n=10, 36%]	germinoma	4	2	CR n=3; SD n=1
	mixed malignant GCT	3 [+1eb]	3	SD n=3; CR n=1
	chorioncarcinoma	[1eb]	-	CR n=1
	malignant teratoma	1	1	SD n=1
Glial Tumors [n=13, 46%]	ganglioglioma	2	1	SD n=2
	pilocytic astrocytoma	2 [+1eb]	3	SD n=2; PD n=1
	low grade astroglial tumor	2	2	SD n=1; PD n=1
	diffuse astrocytoma	1	1	SD n=1
	anaplastic astrocytoma	3	1 [1 shunted]	CR n=1; dead n=2
	H3K27 mutated midline glioma	1	1	dead n=1
	glioblastoma multiforme	1	1	dead n=1
Ependymal Cyst [n=1, 4%]		1	1	*Lost to follow-up*
*total*		25	20 successful ETV: 17/19 (89.5%)	

Abbreviations: *eb* – external biopsy, *SD* – stable disease, *PD* – progressive disease, *CR* – complete remission, *ETV* – endoscopic third ventriculostomy

The initial endoscopic biopsy was followed by a subsequent tumor resection in 13 of 24 patients (54.2%) during the whole follow-up, thereby giving the opportunity for a second tissue sample to reconfirm or adjust the result of the first endoscopic biopsy. The second histological diagnosis matched the result of the first in 12 out of 13 cases which results in a 92.3% accuracy rate of the primary neuroendoscopic biopsy. In one patient, the diagnosis “malignant tumor without specification” after endoscopic biopsy was eventually specified towards a WHO grade III anaplastic astrocytoma after microsurgical resection.

### Complications of primary surgical intervention to establish a diagnosis

The endoscopic biopsy procedures led to complications in 4 of 24 patients. Three transient events occurred (minor morbidity, 12%): deranged sodium levels necessitating medical treatment in two cases and temporary, postoperative strabism in one case. A permanent postoperative morbidity rate of 4% after the primary intervention (major morbidity) was observed in one patient with a postoperative strabism requiring a later operative correction.

### Subsequent tumor treatment (Fig. 2)

After initial histological confirmation, 8 patients (7 newly diagnosed entities and 1 known entity) underwent craniotomy and tumor resection either via a supracerebellar-infratentorial (*n* = 2), a frontal transcallosal-transchoroidal (*n* = 2), or a parietal-transventricular (*n* = 4) approach after a median of 11 days (range 3 to 39 days) after biopsy, because surgery was decided to be the primary and best treatment option (1 papillary tumor of the pineal region, 1 pineoblastoma, 1 mixed malignant germ cell tumor, 1 malignant teratoma, 1 pilocytic astrocytoma, 2 anaplastic astrocytoma, and 1 H3K27-mutated midline glioma). The primary resected mixed germ cell tumor was one of the referred patients with an initial, externally established diagnosis of a teratoma. Only after the gross total resection, the final neuropathological diagnosis of a mixed malignant germ cell tumor was made. For the patient with pilocytic astrocytoma, a spontaneous hemorrhage into the tumor necessitated resection 39 days after initial biopsy. One patient with an anaplastic astrocytoma underwent 2 further resections via altering approaches. Following surgery, the patient with the pilocytic astrocytoma was only observed, while all others received chemotherapy and radiation according to the respective treatment protocol (for malignant teratoma, CWS/MAKEI; for anaplastic astrocytoma, HIT-GBM-D; for pineal anlage tumor, I-HIT-MED Guidance; for papillary tumor of the pineal region, SIOP LGG 2004; for mixed malignant germ cell tumor, SIOP CNS-GCT II).

**Fig. 2:**
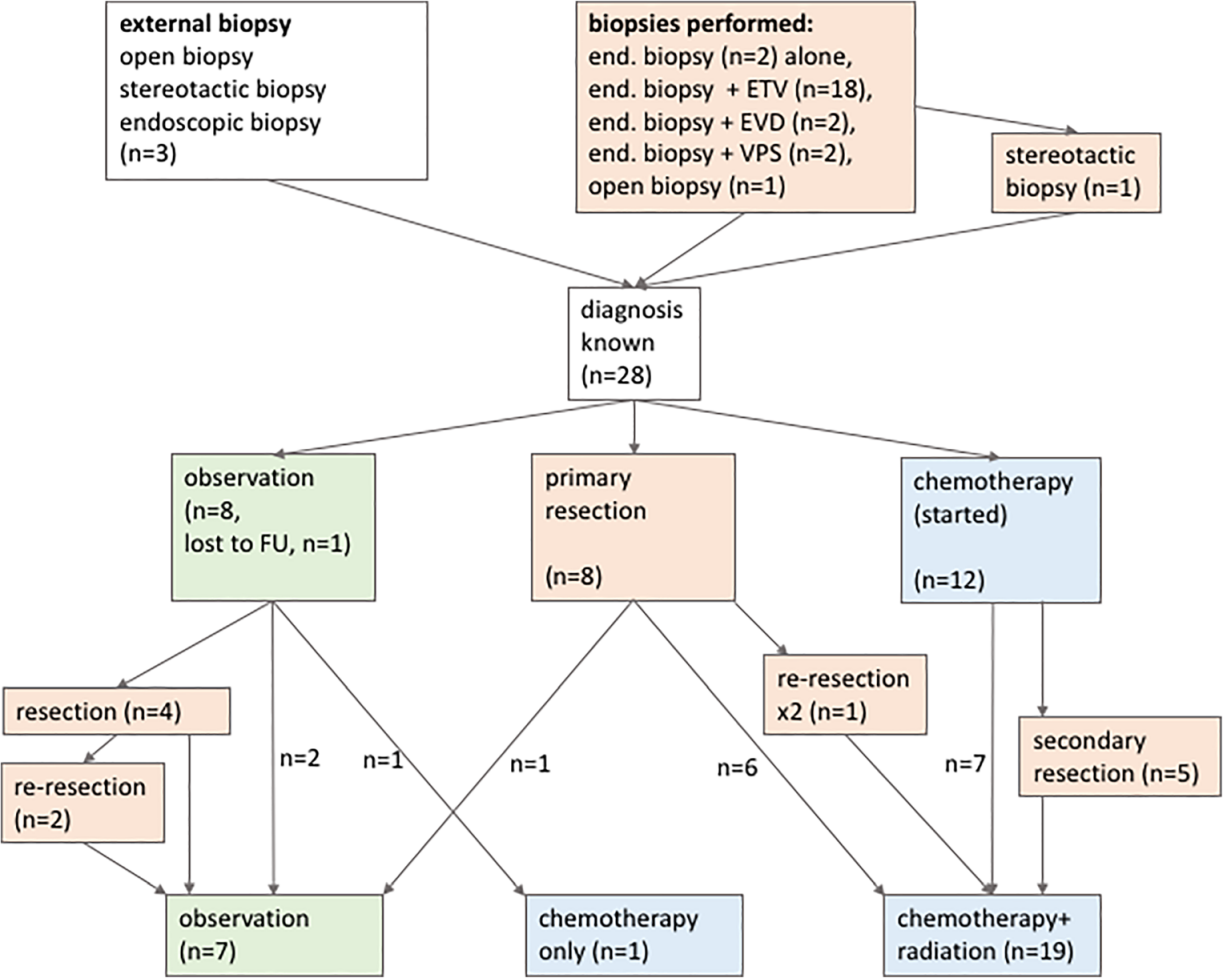
Flow chart of patient treatment regimen. Of the 28 patients with pineal region tumors and confirmed histological diagnosis, only 8 patients (28.6%) underwent a primary resection. Due to the heterogeneity of pineal region tumors, the appropriate treatment should be determined in a multidisciplinary approach

Five patients (one pineal anlage tumor, one choriocarcinoma, and three mixed malignant GCTs) received neoadjuvant chemotherapy after biopsy and underwent later-stage resection via craniotomy after a median of 2 months (range 2 to 3 months) in accordance with the respective treatment protocol. The surgical resection was followed by continuation of chemotherapy and radiation according to the protocol (SIOP CNS-GCT II or I-HIT-MED Guidance). Six patients (4 with germinoma and 2 patients with malignant gliomas—not suitable for resection) started chemotherapy and radiation according to the respective protocol (SIOP CNS-GCT II and HIT HGG 2007). One patient with a disseminated pineoblastoma after primary bilateral retinoblastoma received individualized chemotherapy, i.e., 6 cycles of systemic neuroblastoma protocol cycles (vindesine, cisplatin, etoposide) plus intrathecal etoposide followed by craniospinal irradiation and local boost. The following maintenance therapy consisting of oral trofosfamide, etoposide, and idarubicin was terminated early as the patient suffered from a second local and disseminated relapse 13 months after diagnosis of relapse, treated with temozolomide, irinotecan, sirolimus, and dasatinib.

**Fig. 3:**
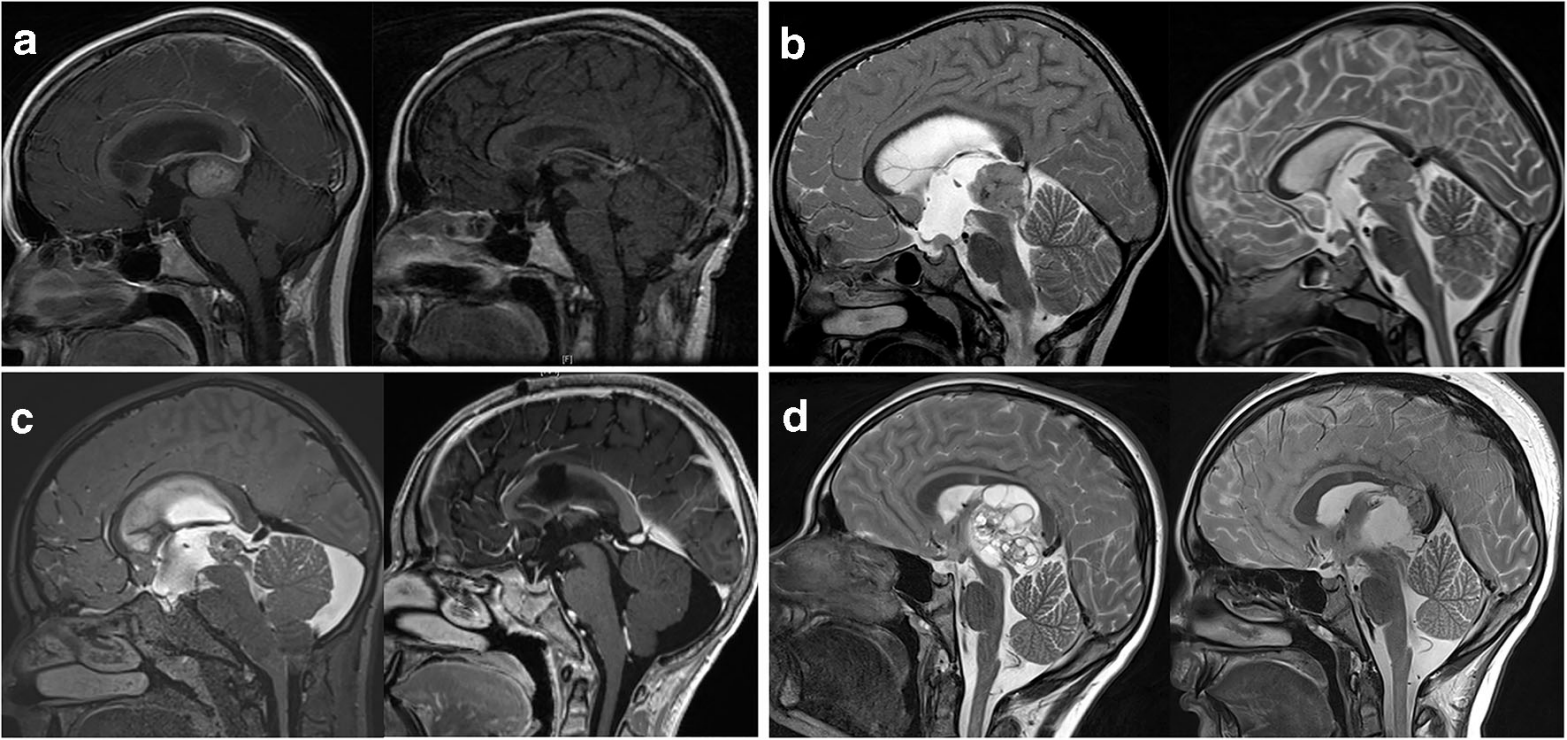
Representative MR images. **a** Papillary tumor of the pineal region, treated with ETV and biopsy, microsurgical supracerebellar infratentorial resection, radiation, and chemotherapy (outcome: stable disease). **b** Disseminated pineoblastoma with involvement of the anterior third ventricular floor, treated with stented ETV and biopsy (right image demonstrating the stent in place though the floor of the third ventricle), radiation, and individualized poly-chemotherapy (outcome: death). **c** Pineoblastoma, treated with ETV and biopsy, neoadjuvant chemotherapy, and transcallosal microsurgical resection, followed by radiation and chemotherapy (outcome: stable disease). **d** Mixed malignant germ cell tumor, transferred after primary biopsy (external hospital), treated by transcortical, transventricular microsurgical resection; radiation; and chemotherapy (outcome: complete remission)

Eight patients did not require any immediate surgical treatment but were recommended to be followed with serial MRIs. One patient with an ependymal cyst was lost to follow-up. Two patients did not require any intervention during an observational follow-up of 34 and 38 months, respectively. Four patients (two pilocytic astrocytoma, one diffuse astrocytoma, one ganglioglioma) underwent craniotomy and secondary partial tumor resection due to documented tumor growth after 2, 3, 55, and 61 months. Two of these patients did not receive any additional treatment after tumor resection. Two patients underwent a second craniotomy and tumor resection because of progressive disease 20 and 44 months after first surgical resection. One patient with a first progression of a low-grade glioma eventually received chemotherapy (SIOP LGG protocol) after 6 months of observation. For exemplary MRIs, see Fig. 3.

### Overall resection rate

Overall, 17 patients of the cohort (13 after endoscopic biopsy, 1 after open biopsy, and 3 after biopsy at a referring hospital) underwent surgical tumor resection during their course of disease: 8 patients as the first intervention after establishing a diagnosis, 5 patients as a staged procedure after neoadjuvant chemotherapy, and 4 patients due to tumor growth on follow-up MRI after observation strategy. As described above, in 3 patients, more than one surgical resection were performed (two patients with 2 resections and one patient with 3 resections) equaling a total number of *n* = 21 surgical tumor resection procedures during the observation period. The surgical approaches for microsurgical resection were parietal-transventricular (*n* = 10), supracerebellar-infratentorial (*n* = 4), and transcallosal-transchoroidal (*n* = 3), as well as a frontal transventricular approach for endoscopic resection. The extent of resection as assessed by postoperative MRI was GTR after 3 procedures, STR after 4 procedures, PR after 9 procedures, and extended biopsy (EB) only after 5 procedures (of those 3 patients after endoscopic approach).

There was no mortality after surgical resection, but 3 patients experienced postoperative gaze palsy (immediate complication rate of 14.3%). In 2 of those patients, the postoperative deficit resolved during follow-up, and in the other patient, it remained unchanged until the patient’s death after 4 months due to rapidly disseminating disease (4.8% permanent complication rate).

### Hydrocephalus treatment during follow-up

Overall, an accompanying non-communicating hydrocephalus was treated with ETV or sETV in 20 patients. Two ETVs failed and necessitated a VP shunt insertion as mentioned above, and one patient was lost to follow-up. The ETV success rate was thereby 89.5% (17 of 19) after a median observation of 3 years and 2 months (range 1 month to 8 years and 11 months).

For two patients who were initially treated with endoscopic biopsy and EVD, a VP shunt was required for permanent CSF diversion. One patient who received an ETV initially needed a VP shunt after resection of an anaplastic astrocytoma. In one patient, a stented ETV needed to be advanced in a VP shunt after 1 year and 1 month. Considering 2 patients who received a VP shunt along with their endoscopic biopsy and two with previously implanted VP shunts, 8 VP shunts (28.6%) were implanted in the entire cohort. Of those patients with a VP shunt, 2 needed additional shunt revisions during a median follow-up of 3 years (range 3 months to 12 years).

### Other neurosurgical procedures during follow-up

One patient with choriocarcinoma who was treated in a multimodal fashion with chemotherapy and radiation therapy developed multiple cavernomas during surveillance and underwent craniotomy for a hemorrhagic cavernoma 5 years and 1 month after initial tumor diagnosis.

### Overall outcome

The median follow-up for all patients was 4.1 years (range 0.1 to 12 years). One patient with a non-tumorous histology (ependymal cyst) was lost to follow-up. The latest clinical status of the remaining 27 patients was CR in 6, SD in 14, PD in 2, and death in 5 patients (Table 1). Death occurred after a median of 13 months (range 3 months to 3 years and 11 months) due to tumor progression.

## Discussion

Pineal tumors account of approximately 2.8–11% of pediatric brain tumors [24]. These tumors display a remarkable diversity and histological heterogeneity. According to the tissue of origin, they are classified as pineal parenchymal tumors (e.g., pineocytoma, pineoblastoma), germ cell tumors, and tumors arising from neuroepithelial tissue [13]. Furthermore, other non-neoplastic lesions like simple cysts or dysplastic lesions (e.g., midline lipoma) can be found in the pineal region. This heterogeneity was again confirmed in our reported pediatric cohort.

The retrospective review of the cohort revealed the following relevant observations with regard to the introductorily formulated questions: (a) neuroendoscopic biopsy of pineal region tumors was an efficient and safe procedure to obtain a histological diagnosis with a diagnostic yield of 95.8% and an accuracy rate of 92.3% and to treat concomitant hydrocephalus, and (b) only a minority of patients (28.6%) underwent primary surgical resection of the pineal lesion once histology was confirmed; the majority of patients was referred for observation or for primary chemotherapy and radiation therapy.

### Neuroendoscopic biopsy and concomitant endoscopic hydrocephalus treatment

The initial presentation of a child with a newly diagnosed pineal lesion remains to be a challenging situation. This is due to the anatomic location in close proximity of various delicate structures like the deep venous drainage system, the structures of the midbrain, the posterior third ventricle, and aqueduct, which accounts for potential surgical morbidity as well as for the common presentation with non-communicating hydrocephalus. Furthermore, the histological variety accounts for the diversity of treatment protocols which may involve a variable combination of surgical resection, chemotherapy, and radiation [6, 21, 25].

Therefore, the therapeutic goals for a child with newly diagnosed pineal region tumors are threefold: treatment of the accompanying hydrocephalus, establishing a histological diagnosis, and finally deciding on the optimal treatment protocol for the pineal tumor. Treatment of hydrocephalus and the associated elevated intracranial pressure can consist of extracranial CSF diversion or establishing an internal communication by an ETV [9, 20]. The confirmation of a histological diagnosis can also be obtained by an endoscopic procedure, while stereotactic biopsy represents the possible alternative. However, a stereotactic biopsy does not resolve any coexisting hydrocephalus, thus requiring additionally either an EVD, insertion of a VP shunt, or an ETV in a second surgical intervention [18, 31]. The success rates of stereotactic biopsy are reported to be high with a diagnostic yield of 93.7 to 100% [3, 18, 31].

In contrast, an endoscopic approach allows procurance of a tissue sample and treatment of hydrocephalus during one surgical procedure. The diagnostic yield of an endoscopic biopsy was reported to be at 81.1 to 100% [2, 3, 28, 33, 37]. In addition to this diagnostic yield rate, the rate of accordance of the first histological result after biopsy with the second histological results after a later resection (or repeated biopsy) represents the diagnostic accuracy rate [4]. The accuracy rate is especially of importance in pineal tumors because of their known heterogeneity with differing histological entities, but also known heterogeneity within one pineal lesion. A biopsy taken from one region might not necessarily display all tumor characteristics, and areas with a more aggressive behavior might be missed, resulting in selection of a non-appropriate treatment protocol. The reported diagnostic accuracy for endoscopic biopsies is in the range of 50–78.6% [2, 14]. Especially, the subgroup of germ cell tumors includes mixed pathologies with areas of differing biological behavior. Accordingly, a low diagnostic accuracy of 50% (3 of 6) after endoscopic biopsy was reported for this entity [14]. Therefore, a risk remains that the most malignant portion might be missed by first biopsy and either repeated biopsy or surgical resection of residual tumor after initial non-surgical therapy might become necessary. This option is addressed in the multimodal treatment protocol for germ cell tumors [6].

The diagnostic accuracy for stereotactic biopsy is reported to be 100% in one series [18]. Two analyses comparing endoscopic vs. stereotactic biopsies stated that the stereotactic technique is “safer and more effective” than the endoscopic approach and recommended therefore a combination of an endoscopic procedure for hydrocephalus treatment and a stereotactic biopsy to obtain tissue for diagnosis [3, 4]. However, the analysis of our series with a 95.8% diagnostic yield, a 92.3% accuracy rate, and only 4.2% permanent morbidity does not support this conclusion. This is in keeping with a review of key data of recently published studies comparing endoscopic and stereotactic biopsies of pineal lesions (Table 2). The advantage of the endoscopic approach avoiding a second surgical procedure to treat hydrocephalus undoubtedly favors the endoscopic approach. The 89.5% patency rate of the performed ETVs after a median of 3 years and 2 months in our series underlines the potential of this procedure to efficiently treat the non-communicating hydrocephalus during the same surgical intervention. Increasing experience in endoscopic procedures makes this approach extremely compelling, as both surgical targets, namely the hydrocephalus and the procurement of tissue, are addressed in one intervention through the same frontal entry point. A crucial prerequisite for the combined biopsy and third ventriculostomy via an endoscopic approach with a rigid endoscope is the meticulous planning of the burr hole according to the trajectories to the floor of the third ventricle and the pineal region tumor. In this cohort, we determined the angle bisector of the ideal trajectories to both regions of interest in order to identify the ideal point of entry at the skull surface [15, 16], a method that has been validated in later studies [8]. The use of a flexible endoscope for biopsy and ETV or a combination of flexible and rigid endoscopes could possibly minimize the tissue manipulation at the level of the foramen of Monro [33]. However, in the presented series, no adverse effects or relevant complications attributable to damaged neuronal structures at the foramen of Monro were seen. In general, the complication rate of this series is considerably low. The transient derangement of sodium levels in two patients, both with bilocular germ cell tumors who presented initially with hydrocephalus and were affecting the infundibulum and the pituitary stalk, is likely to be caused by the involvement of the hypothalamus-pituitary axis. Two patients experienced disturbed gaze and strabismus, which was temporary in one, but persisting in the other (glial tumor in subependymal location), resulting in a permanent complication rate of 4.2%. This is in line with previously reported complication rates (Table 2). In view of the presented patient series and the reviewed literature, simultaneous ETV and endoscopic tissue sampling has evolved to be our intervention of first choice. Stereotactic biopsy is reserved for patients with already treated hydrocephalus and non-diagnostic endoscopic biopsy (4.2% in this series).

**Table 2:** Literature review of pineal tumor biopsy

	Author	Number of patients	Age	Hydrocephalus treatment	Possible biopsy	Diagnosis rate	Mortality	Morbidity	Subsequent resection	Accuracy of first diagnosis*	Comment
Endoscopic biopsy	Morgenstern (2011)	15	Mean, 37 years (range 6–71)	100% (HC as inclusion criterion)	NS	86.7%	0%	0%	60% (9 of 15)	NS	
	Roth (2015)	6	Range, 3.5–53 years	100% (HC as inclusion criterion)	100%	100%	16.7% (1 of 6)	NS	50% (3 of 6)	66% (2 of 3)	Tumors of the posterior third ventricle (pineal, thalamic, tectal)
	Ahmed (2015)	48	Mean, 26 years (range 2–68); 18 patients less than 18 years	100% (HC as inclusion criterion)	95.8% (46 of 48)	84.8% (39 of 46)	None	21.7% (10 of 46)	50% (24 of 48)	78.6% (22 of 28)	
	Kinoshita (2017)	21	Median, 14 years (range 8–51)	17 of 21 (80.9%)	100%	100%	0%	0%	28.6% (6 of 21)	50% (3 of 6)	Germ cell tumors only
	Oertel (2017)	48 (pineal region) of 130 patients	Range, 4 months–82 years; 29 less than 15 years	NS	100% (48 of 48)	91.6% (44 of 48)	NS	NS	NS	NS	Series with differing biopsy sites (pineal region among others—third, fourth, and lateral ventricles; aqueduct; thalamus; pineal; and clivus)
	Torres-Corzo (2018)	48 (27 pediatric patients, 21 adults—among them, 11 + 10 in the thalamic + pineal region)	Mean, 20.4 years	79.2% (38 of 48)	NS	83.3%		0% major			Flexible neuroendoscopy, rates given only for all intraventricular locations together
	Balossier (2015)					Mean of 81.1%	0.4%	21% minor, 2% major			Meta-analysis of publications in 1970–2013
	Present study	24	12 years and 4 months (range 5 months to 17 years and 7 months)	91.7% (22 of 24)	100%	95.8% (23 of 24)	0%	12.5% (transient, minor), 4% permanent (major)	54.2% (13 of 24)	84.6% (11 of 13)	Subgroup of patients with endoscopic biopsy only (24 of 28)
Stereotactic biopsy	Balossier (2015)					93.7%	1.3%	6.4% minor, 1.6% major			Meta-analysis of publications in 1970–2013
	Lefranc (2011)	88	Median, 30 years (range 2–74)	72.5% (prior ETV or shunt procedure)	100%	99%	0%	6%	26.1% (23 of 88)	100% (23 of 23)	
	Quick-Weller (2016)	14	Median, 39.5 years	6 + 2 of 14	100%	100% (of which 7.1% was necrosis)	NS	NS	21.4% (3 of 14)	NS	

*NS* not specified

*As confirmed by either resection or repeated biopsy

### Biopsy vs. primary resection without biopsy

If a child presents with a newly diagnosed pineal tumor, a decision has to be made whether to obtain a biopsy first or to plan a surgical resection via a craniotomy. As an exception, in the clinical setting of elevated CSF concentration of AFP and/or β-HCG in the combination with a typical radiological appearance of a germ cell tumor on MRI, histological confirmation can be omitted and treatment initiated without biopsy. Ongoing progress in the field of microsurgery has subsequently lowered complication rates of these complex interventions. There are recent reports about primary microsurgical resection of pineal tumors with low experienced complications and improved function without prior biopsy [10, 12, 17, 29]. In some of those series, no tumor of the germ cell type group was specifically mentioned [10, 17]. Still, permanent “minor” morbidity—double vision, hypacusis, and cerebellar symptoms—was reported for 13% of patients [10]. The low prevalence of germ cell tumors in this presumably adult patient series is in contrast to the 10 of 28 tumors (35.7%) from this subgroup in our presented series. After establishing a histological diagnosis, only 28.6% (8 of 28) of our patient cohort had to undergo primary surgical resection as the best possible treatment option as guided by the German pediatric neuro-oncological network (HIT) recommendations [11]. For the remaining 71.4%, either initiation of chemotherapy ± radiation or observation is currently recommended depending on the tumor type. Thus, a majority of patients would have been exposed to an inadequate risk of an invasive procedure if the tumor had been primarily resected. Even after a median observation of 4 years and 1 month, only 60.1% (17 of 28) of our patient cohort needed surgical resection. This is in line with reported rates of necessary surgical resection after primary endoscopic or stereotactic biopsy with 21–60% [2, 14, 23, 31, 33]. Based on the data of this presented pediatric series and on the reviewed publications, primary resection, without preceding biopsy, cannot be recommended in children. As general guideline, the German HIT–based indication for resection is given in low-grade gliomas, teratoma, resectable high-grade glioma, and papillary tumor of the pineal region. The more recent established neoadjuvant chemotherapy concept is followed by secondary resection and would be suitable in mixed cell or malignant germ cell tumors, pineoblastoma, and tumors of the pineal region in infancy in order to limit surgical morbidity. No surgical indication would be given in pure germinoma and non-resectable-high grade or H3K27M glioma. Apart from this guideline, indication for pineal tumor surgery is also based on the local mass effect and clinical condition of the patient, respectively.

The surgical resection achieved a combined gross total or subtotal resection rate of 33% and a partial resection rate of 43% in this cohort. The complication rate of 14.3% for surgical resection procedures is comparable to recently published series with rates between 9.7 and 24% [1, 10, 19, 30, 38]. Thereby, long-term morbidity for the patients could be avoided in order to preserve long-term quality of life. Thus, resection strategy and its applied risk in this cohort are based on the feasibility of resection mainly defined by the tissue texture and its bleeding characteristic, which was set in the context to the availability and application of the described multimodal therapeutic approach. For a few patients, a pure endoscopic resection of tumor parts has been applied in this series, mainly for patients with residual disease after neoadjuvant therapy and for intraventricular parts of intrinsic gliomas. Although currently the achievable degree of resection with a purely endoscopic technique may still be limited, significant improvement with advancement of endoscopic ultrasound aspirators and guiding technology have been described and are further developed to minimize the surgical approach and possibly the risk of a procedure [7, 26, 27, 36].

### Limitation

The analysis of our presented patient series might be less representative due to the relatively limited group size because of the low incidence of pineal region tumors even in a large volume pediatric neurosurgical unit. Furthermore, the heterogeneity of the treated pineal region tumors and small numbers of each histological entity will not allow significant conclusions about the long-term prognosis and survival.

## Conclusions

The presented data constitute a series of pediatric patients with different subtypes of pineal region tumors presenting with non-communicating hydrocephalus in the majority of cases. We have shown that endoscopic tumor biopsies are safe and yield high diagnostic accuracy rates that are comparable to previously reported results of stereotactic biopsy. The endoscopic approach offers, however, the simultaneous treatment of the non-communicating hydrocephalus. Considering the fact that less than a third of patients needed primary surgical resection once the histological diagnosis was confirmed, our results suggest that a primary biopsy of pineal lesions should precede attempted surgical resection in children. Primary surgical resection without biopsy would expose a significant proportion of patients of this series (71.4%) to an unnecessary risk.

It will be necessary to perform prospective multicenter collection focusing on surgical strategies of these tumors in order to acquire more solid data.
